# Cold hearts and dark minds: a systematic review and meta-analysis of empathy across dark triad personalities

**DOI:** 10.3389/fpsyt.2025.1546917

**Published:** 2025-02-27

**Authors:** Meenakshi Shukla, Niti Upadhyay

**Affiliations:** ^1^ Department of Psychology, University of Allahabad, Prayagraj, India; ^2^ Department of Psychology, Banaras Hindu University, Varanasi, India

**Keywords:** cognitive empathy, affective empathy, Dark triad, systematic review, meta-analysis, narcissism, psychopathy, Machiavellianism

## Abstract

**Introduction:**

This systematic review and meta-analysis explored cognitive and affective empathy differences across Dark Triad traits—Narcissism, Machiavellianism, and Psychopathy.

**Methods:**

Registered on PROSPERO and following PRISMA guidelines, PubMed, Scopus, Science Direct, ProQuest, and Google Scholar were searched for studies published until June 2024. Risk of bias was evaluated using Egger’s test and Rank correlation test, along with risk-of-bias plots (Robvis) for quality assessment.

**Results:**

Fourteen studies (N = 5,328) met the inclusion criteria. Meta-analysis showed Narcissism was negatively associated with affective empathy (r= -.134, p<.05) but not significantly linked to cognitive empathy (r= .061, p= .215), while Machiavellianism had a significant negative correlation with both cognitive (r= -.089, p<.05) and affective empathy (r= -.291, p<.0001). Psychopathy demonstrated the strongest negative association with affective empathy (r= -.347, p<.0001). Moderate-to-high heterogeneity was found across all analyses (I^2^ range: 40.56% - 94.03%).

**Discussion:**

This review underscores differential empathy profiles across Dark Triad traits, with significant affective empathy deficits in Psychopathy and Machiavellianism and the complex role of cognitive empathy in Narcissism and Machiavellianism. Further research should examine situational and subtype -specific factors influencing empathy in Dark Triad traits to enhance theoretical understanding and inform interventions.

**Systematic review registration:**

https://www.crd.york.ac.uk/prospero/display_record.php?ID=CRD42024559533, identifier CRD42024559533.

## Introduction

Empathy is a complex, multifaceted concept that involves understanding and sharing the emotions of others (1). Wispé (2) described empathy as an active effort to comprehend the positive and negative experiences of others, a process believed to prevent exploitative and harmful behaviors while encouraging prosocial actions (3; see review, 4). Research on empathy has led to distinguishing between two types: cognitive and emotional/affective empathy. Dual-process models suggest that cognitive empathy, involving perspective-taking and understanding others’ mental states, is a rational process, while affective empathy, involving emotional resonance, is more automatic and less cognitively demanding (5, 6). Neurological evidence supports this model, showing that various cognitive empathy tasks, such as theory of mind and perspective taking, involve distinct pre-frontal brain activations (6), are closely related to executive functioning [meta-analysis, (7)], and require more mental resources compared to emotional empathy (8–10).

Generally, cognitive empathy entails understanding others’ thoughts and feelings without necessarily having an emotional response whereas emotional empathy involves feeling emotions in response to others’ emotional experiences or expressions (11). However, there is debate among researchers about whether this distinction is meaningful, which concepts fall under each category, and whether some concepts should be considered empathy at all (12, 13). Cognitive empathy encompasses perspective taking and the ability to understand another’s situation by imagining their feelings (online simulation) (14). Affective empathy, in contrast, is demonstrated through an individual’s emotional resonance with someone else, encompassing the ability to vicariously experience another’s emotions (emotional contagion), react to others’ emotional signals (proximal responsivity), and engage with emotional cues in immersive contexts (peripheral responsivity) (14). Some researchers categorize affective empathy into empathic concern, emotional contagion, and personal distress (15). These subtypes differ in their focus on the self-versus-others. Empathic concern is other-oriented, while personal distress involves self-focused emotional reactions. Emotional contagion involves a mix of self- and other-focus, mirroring others’ emotions. Self-report measures of empathic concern and personal distress are generally negatively correlated (16) and relate differently to prosocial behavior (17). A meta-analysis found that self-reported empathy and empathic abilities do not always align, as self-reported empathy reflects the motivation to empathize rather than the actual skill (18).

Some researchers have explored how perspective taking and predicting others’ mental states can be misused in contexts like cheating, manipulation, or interpersonal exploitation (19–21). Clinical studies often highlight a deficit in affective empathy in psychopathy, while cognitive empathy remains intact (22–26), though a recent meta-analysis (27) indicated deficits in both cognitive and affective empathy in psychopathy, suggesting ambiguity in the existing literature. Researchers have examined whether and how perspective taking and understanding others’ mental states can be misapplied in situations such as cheating, manipulation, or exploitation (e.g., 19–21). In the last two decades, researchers’ interests have been quite focused on how empathy deficits (or enhancement) manifest among individuals with Dark Triad traits.

Empathy plays a pivotal role in fostering prosocial behaviors by enabling individuals to understand and resonate with the emotional states of others. However, empathy is not a universal experience, as significant variability exists across individuals, particularly those exhibiting traits associated with the Dark Triad. These socially aversive personality traits are characterized by behaviors such as emotional detachment, manipulation, and self-centeredness, which inherently conflict with the foundational principles of empathy. Understanding how cognitive and affective empathy are uniquely impaired across these traits is critical for advancing theoretical models and addressing the behavioral and interpersonal challenges posed by these individuals.

The Dark Triad comprises three interconnected personality traits that are socially and interpersonally detrimental: subclinical Narcissism, Machiavellianism, and subclinical Psychopathy (28). From here forward, the use of the terms Narcissism and Psychopathy would be used to refer to subclinical Narcissism and subclinical Psychopathy, respectively. These traits share a common core of manipulativeness, emotional detachment, and egocentricity (28), but differ in their manifestations and their relationships with empathy.

Narcissism is marked by egocentricity, a sense of entitlement, and grandiose thinking (28) and presents a complex relationship with empathy. While narcissistic individuals often exhibit reduced affective empathy due to their self-centered nature, evidence suggests that their cognitive empathy can remain intact or be even enhanced, enabling them to navigate social situations strategically for self-serving purposes (29). For instance, narcissists may engage in perspective-taking to achieve their goals, even as their emotional resonance with others remains limited. However, individuals with higher scores of pathological grandiosity demonstrate greater emotional intelligence and empathy (30) and cognitive empathy appears to be intact or even enhanced in Narcissism (31, 32).

In contrast, Machiavellianism is characterized by an exploitative, deceitful, and cynical nature (33). It reflects a cold, calculated approach to interpersonal relationships, driven by manipulation and deceit (34). Individuals high in Machiavellianism exhibit consistent deficits in both cognitive and affective empathy (31, 32, 35), aligning with their emotionally detached and exploitative tendencies. Thus, unlike narcissists, whose cognitive empathy may support their interpersonal goals, Machiavellians tend to lack both the emotional resonance and perspective-taking abilities needed for genuine prosocial engagement.

Psychopathy, the third trait in the Dark Triad, is defined by a profound lack of emotional resonance, impulsivity, and callousness (36). Among the three traits, psychopathy demonstrates the strongest and the most consistent empathy deficits, particularly in affective empathy (22). These individuals are often incapable of sharing or understanding others’ emotional experiences, which contributes to their antisocial and aggressive behaviors. While cognitive empathy may occasionally be intact (e.g., 22–26), it is inconsistently applied and often serves manipulative ends rather than fostering meaningful social connections.

Impaired empathy is a central feature of the Dark Triad, particularly linking psychopathy and Machiavellianism (31, 32). Research consistently indicates a negative association between both affective and cognitive empathy and psychopathic traits, even when accounting for the influence of the other two traits (31, 32, 35). For Machiavellianism and psychopathy, deficits in emotional empathy are more pronounced, whereas narcissism shows no significant correlation with emotional empathy (35). Interestingly, psychopathy alone predicts deficits in both cognitive and affective empathy, though some clinical studies suggest that cognitive empathy remains intact in psychopathy, with affective empathy being the primary deficit (22–26).

The relationship between narcissism and empathy is more complex. While some studies report no significant association (37), others suggest that individuals with pathological grandiosity may exhibit higher emotional intelligence and empathy (30). Cognitive empathy in narcissism, in particular, appears intact or even enhanced, potentially enabling strategic social interactions (31, 32). Further complicating the literature, recent findings from latent profile analyses reveal subgroups within the Dark Triad, including a “Dark Empath” profile characterized by high empathy and fewer negative outcomes such as aggression and impaired well-being compared to the traditional Dark Triad profile with low empathy (29).

The literature on the Dark Triad traits and their relationship with empathy reveals both recurring patterns and significant inconsistencies. These inconsistencies in how empathy deficits manifest across the Dark Triad traits underline the need for a comprehensive synthesis to clarify the distinct cognitive and affective empathy profiles associated with each trait. Psychopathy is consistently associated with pronounced deficits in affective empathy, yet findings on cognitive empathy are contradictory: while some studies suggest intact or even heightened cognitive empathy that facilitates manipulative behaviors (38, 39), others report impairments in understanding others’ perspectives (22, 40). Similarly, Machiavellianism is broadly linked to deficits in both cognitive and affective empathy, aligning with its emotionally detached and exploitative nature (41, 42); however, evidence indicates that Machiavellians may strategically deploy cognitive empathy when it serves their goals (43). In contrast, narcissism demonstrates the most variability, with grandiose narcissists often exhibiting intact or enhanced cognitive empathy for self-promotion, while vulnerable narcissists show pervasive deficits in both empathy domains (31, 44). Emerging constructs like “Dark Empaths”—individuals combining high cognitive empathy with dark traits—further challenge the assumption of universal empathy deficits across the Dark Triad (29). These inconsistencies are exacerbated by methodological variability, with studies relying on divergent tools such as the Interpersonal Reactivity Index and the Toronto Alexithymia Scale, which may inadequately distinguish between cognitive and affective empathy (40, 42).

To enhance clarity in understanding the role of empathy in Dark Triad traits, this systematic review and meta-analysis aimed to answer the following question: How do cognitive empathy and affective empathy differ among the Dark Triad traits of Narcissism, Machiavellianism, and Psychopathy? Given that past research has yielded mixed findings on this relationship, this study sought to systematically summarize published studies to examine how the two forms of empathy (cognitive and affective) interact with each Dark Triad trait.

Beyond assessing overall relationships, this meta-analysis also conducted subgroup analyses based on measurement scales, given the diversity in tools used to assess both Dark Triad traits and empathy. These analyses were essential for evaluating whether differences in measurement approaches contribute to variations in effect sizes. Additionally, we initially intended to conduct gender-wise and country-wise subgroup analyses to explore potential cultural and demographic influences on these relationships. However, due to insufficient reporting of gender-specific and country-specific effect sizes in many studies, these analyses were not feasible.

By synthesizing literature on empathy profiles across Dark Triad traits, this study contributes to refining theoretical models of empathy by clarifying whether cognitive empathy (understanding others’ emotions) and affective empathy (feeling others’ emotions) differentially relate to Narcissism, Machiavellianism, and Psychopathy. Additionally, understanding trait-specific empathy deficits can offer insights into mechanisms underlying manipulative, exploitative, and antisocial behaviors, with implications for workplace conflict resolution, leadership strategies, and psychological interventions.

## Methods

### Protocol registration

The procedure for this systematic review and meta-analysis was registered on the International Prospective Register of Systematic Reviews database (PROSPERO) under the Ref. No.: CRD42024559533, and the presentation of findings of this study comply with the Preferred Reporting Items for Systematic Reviews and Meta-Analyses (PRISMA).

### Search strategy

Studies for potential inclusion in this systematic review and meta-analysis were identified following a search of these electronic databases: Scopus, PubMed, Science Direct (last assessed: September 30, 2022), ProQuest, and Google Scholar (last assessed: July 13, 2024). Only studies in English language and published until June 2024 were considered. The search dates were from 22-06-2024 to 30-07-2024. The search string used was: (“Dark Triad” OR “Psychopath*” OR “Machiavellian*” OR “Narcissis*”) AND (“Empath*” OR “Cogni* Empath*” OR “Affect* Empath*”). The search sting was adjusted to suit the wildcard and truncation of the various databases searched, where necessary.

### Inclusion and exclusion criteria

The inclusion criteria for selecting studies were: 1) Studies conducted on normal healthy participants; 2) Studies that were experimental, longitudinal, cross-sectional, or cohort studies; 3) Studies published in English language; 4) Studies published until June 2024; and 5) Studies stating the correlation of empathy (cognitive/affective/both) and Dark Triad (Narcissism/Machiavellianism/Psychopathy/all three). The exclusion criteria therefore were: 1) Studies involving patient groups; 2) Studies that were reviews (systematic/narrative/scoping/etc.), meta-analyses, case studies, conference abstracts, or opinion pieces; and 3) Studies published in languages other than English.

### Data extraction

Two reviewers (M.S. & N.U.) independently selected the articles and reviewed the title, abstract, and full text of each article to determine whether they met the inclusion criteria. Any disagreement regarding the inclusion/exclusion of a study was resolved through mutual discussions. Then both the reviewers independently extracted the data from included studies. The extracted data was tabulated under the following heads: S. No., Authors and year of publication, Study design, Sample size & location, Measures of Dark Triad, Measures of empathy, Outcomes of the study, Correlations of AE (Affective Empathy) with Dark Triad traits, and Correlations of CE (Cognitive Empathy) with Dark Triad traits. Both the reviewers (M.S. & N.U.) independently extracted the data and in the event of a disagreement, resolution was reached through mutual discussions. For studies that met the inclusion criteria but had missing information in any of the specified data categories listed above, the corresponding author was contacted via email to request the necessary details.

### Publication bias and quality assessment

Publication bias was checked using Egger’s test (45). A significant Egger’s test is considered indicative of high publication bias. Additional analyses for publication bias, including the trim-and-fill method and funnel plot asymmetry test, were carried out for further assessment (46). To evaluate study quality, we generated risk-of-bias plots using the ‘robvis’ software (47). A weighted summary plot visually displayed the amount of information for each bias judgment. A comprehensive risk-of-bias assessment for all studies, which included both overall and domain-specific evaluations, was illustrated using a traffic light plot. To ensure an unbiased quality assessment, the two authors (M.S. & N.U.) independently reviewed each study for their quality and arrived at a consensus with mutual discussions in case of any disagreement.

## Measures

### Empathy measures

#### Empathy quotient scale

Both affective and cognitive empathy were mostly measured using the Empathy Quotient Scale (EQ) or its shorter version (by six studies). While Schimmenti et al. (48) and March (49) have used the EQ scale developed by Lawrence et al. (50), where Schimmenti et al. (48) used the Italian version of this scale developed by Preti et al. (51), Sest and March (52) as well as Wai and Tiliopoulos (32) used the EQ scale developed by Baron-Cohen and Wheelwright (53). The scale by Baron-Cohen and Wheelwright (53) includes 11 items each assessing cognitive and affective empathy while the one developed by Lawrence et al. (50) comprises 17 items measuring affective empathy and 14 items assessing cognitive empathy. Higher scores in both these scales indicate higher levels of cognitive and affective empathy. Sparavec et al. (54) used the short version of EQ developed by Wakabayashi et al. (55) containing 22 items. Similarly, Andrew et al. (41) used 15 items from the short form of EQ scale developed by Muncer and Ling (56) along with 16 filler items from the EQ scale developed by Baron-Cohen (57).

#### Basic empathy scale

The second most-commonly used scale among these studies was the Basic Empathy Scale (BES; 58), which contains 11 items for measuring affective empathy and 9 items for measuring cognitive empathy. The scale was used by Jonason and Krause (35), Pajevic et al. (42), and Puthillam et al. (59). As per a meta-analytic study (60), for cognitive empathy, the mean Cronbach’s α across studies was .81 (95% CI: .77–.85), indicating good internal consistency Similarly, for affective empathy, the mean Cronbach’s α was also .81 (95% CI: .76–.84), reflecting good reliability. The authors of this meta-analytic study suggested that while the BES is suitable for use in general population groups, its application for clinical diagnostic purposes is limited.

#### Questionnaire of cognitive & affective empathy

Two studies (14, 40) used the Questionnaire of cognitive & affective empathy (QCAE) (61) containing 12 items for affective empathy and 19 for cognitive empathy assessment. The scale has adequate reliability and demonstrated convergent validity by showing strong positive associations with BES scores for cognitive and affective empathy (61). The scale also has adequate construct validity (61).

#### Emotional Empathy Scale

To measure affective empathy, one study used the Emotional Empathy Scale (EES, 33 items; 62). The scale has been reported to have high reliability and discriminant validity (62).

#### Affective and cognitive measure of empathy

This scale comprises 12 items (63). The scale has good internal consistency (>.85; 63) and convergent validity (see review, 64).

#### Toronto empathy questionnaire

This scale comprises 16 items (65). The TEQ has adequate internal-consistency reliability (α = 0.79 to 0.87), temporal stability, as well as convergent validity (see review, 64).

#### Interpersonal reactivity index

The Chinese version of the Interpersonal Reactivity Index (IRI; 66) containing 22 items was used by Zhang et al. (67). The scale has been translated into other language versions as well, such as Spanish, Portuguese (Portugal and Brazil), French, and Russian. The scale has good internal-consistency reliability and temporal stability and its 8 to 12 weeks test-retest reliability is excellent. The scale has adequate factorial and convergent validities (see review, 64).

### Dark triad measures

#### Short dark triad

Among the selected studies, the most-widely used measure to assess the Dark Triad traits was the 27-item Dark Triad of Personality – Short Version (SD3, 36). This scale was used by four studies to assess the Dark Triad traits. Though considered an efficient, reliable, and valid measure of Dart Triad personality (36), recent studies report weak convergent validity and poor discriminant validity for the scale, though test-retest reliability was high (68).

#### Dark triad dirty dozen

The next most-utilized measure of Dark Triad (used by three studies) was the Dark Triad Dirty Dozen (DTDD; 69); however, Fan et al. (43) used it to assess only Machiavellianism. The DTDD has been reported to have adequate internal consistency in terms of Cronbach´s alpha and Omega coefficients, convergent validity, discriminant validity, and criterion-related validity (70).

#### Narcissistic personality inventory

Certain trait-specific measures were also used, such as the Narcissistic Personality Inventory (NPI; 71) used to measure Narcissism (in 3 studies). The NPI demonstrates strong reliability, with a test-retest correlation of r = .81 (72), and internal consistency ranging from acceptable to excellent (α = .68-.87; 73, 74). It has good construct validity (75).

#### Mach-IV

Mach-IV (34) was used to assess Machiavellianism. The Mach-IV is a 20-item self-report questionnaire designed to assess Machiavellianism, which encompasses manipulative, deceitful, exploitative, and distrustful tendencies. It evaluates three distinct dimensions: (1) interpersonal tactics; (2) cynical views of human nature; and (3) utilitarian morality. Though it is considered a reliable and valid measure (76), some researchers (e.g., 77) find its use problematic.

#### Levenson self-report psychopathy scale

This scale (78) was used to measure Psychopathy (3 studies). The measure evaluates two dimensions: primary psychopathy, which reflects emotional traits associated with psychopathy, and secondary psychopathy, which pertains to behavioral aspects linked to a psychopathic lifestyle. It demonstrates adequate internal consistency, with Cronbach’s alpha values of .82 for primary psychopathy and .63 for secondary psychopathy (78).

#### Narcissistic personality inventory for children

One study used the Narcissistic Personality Inventory for Children (NPIC; 79). The NPIC is a 40-item measure adapted from the Narcissistic Personality Inventory (NPI) for use with adolescents. Participants choose between paired statements (e.g., “It scares me to think about me ruling the world” vs. “If I ruled the world, it would be a better place”) and rate their selection as “sort of true” or “really true,” with items scored on a 0 to 3 scale. The NPIC provides both total and subscale scores and includes two composite scales: Adaptive Narcissism (Authority and Self-Sufficiency subscales) and Maladaptive Narcissism (Exhibitionism, Entitlement, and Exploitativeness subscales) (79).

#### Self-report Psychopathy Scale-Short form

This scale by Paulhus et al. (80) is a 29-item self-report measure derived from the 64-item Self-Report Psychopathy Scale- 4th edition (SRP-4; 80). It assesses four facets of psychopathy: interpersonal (INT), affective (AFF), lifestyle (LIF), and antisocial (ANT). Each subscale, except ANT, includes seven items rated on a 5-point Likert scale (1 = strongly disagree to 5 = strongly agree), with facet scores calculated by summing item responses.

### Statistical analyses

The statistical approach used to examine the relationship between two continuous variables across multiple studies is known as meta-correlation, or more specifically, meta-analysis of the correlation coefficient r (81). For each study, the correlation coefficient between the two variables was combined using meta-analytic techniques to derive a general estimate of the strength of the relationship (81). To standardize the effect sizes for each correlation coefficient, Fisher’s r-to-z transformation (81) was used. This transformation normalizes the correlation coefficients, allowing for more accurate estimation of the overall effect size and standard error (81). Using these standardized effect sizes, the overall correlations between the relevant variables in our meta-analysis were calculated, i.e., affective and cognitive empathy were correlated individually with each of the three Dark Triad traits of Narcissism, Machiavellianism, and Psychopathy.

Heterogeneity was evaluated primarily through the use of the I-squared (I²), H statistic, tau (τ), and tau-squared (τ²) measures (82, 83). The I² statistic measured the percentage of total variation in effect sizes attributable to heterogeneity rather than random chance and values of 25%, 50%, and 75% for I² were interpreted as indicating low, moderate, and high levels of heterogeneity, respectively (83). The H statistic assessed how much influence individual studies had on the overall meta-analysis results (83). The tau (τ) statistic represented the variance between studies in effect sizes, with higher τ values indicating greater heterogeneity (82). Tau-squared (τ²) was the estimated variance of the true effect sizes across studies after adjusting for sampling error (82).

## Results

**Figure 1:**
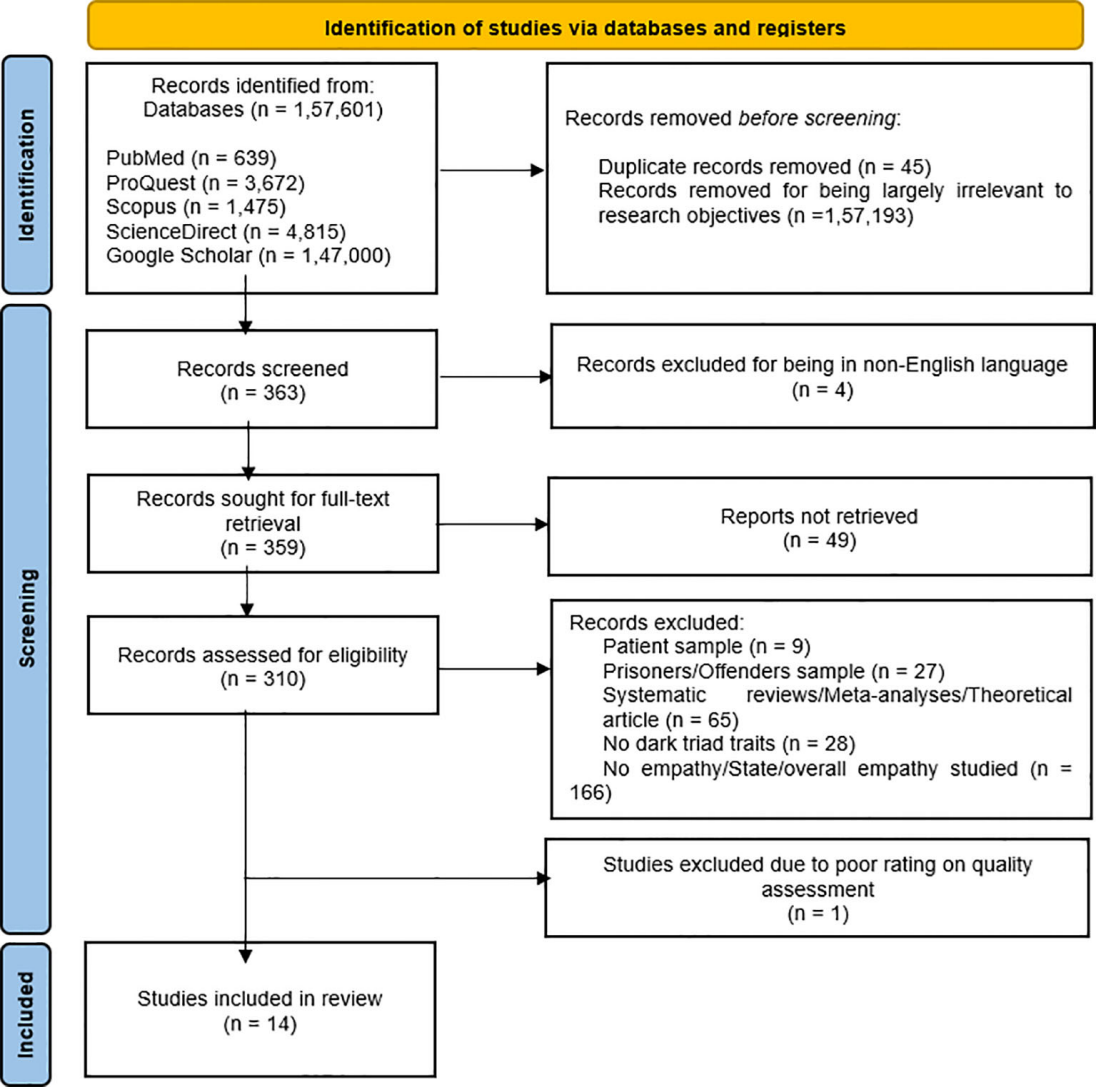
Illustration of the study search and selection process.

The procedure used in the shortlisting of studies has been illustrated in the PRISMA flowchart depicted in **Figure 1**.

### Quality assessment

**Figure 2:**
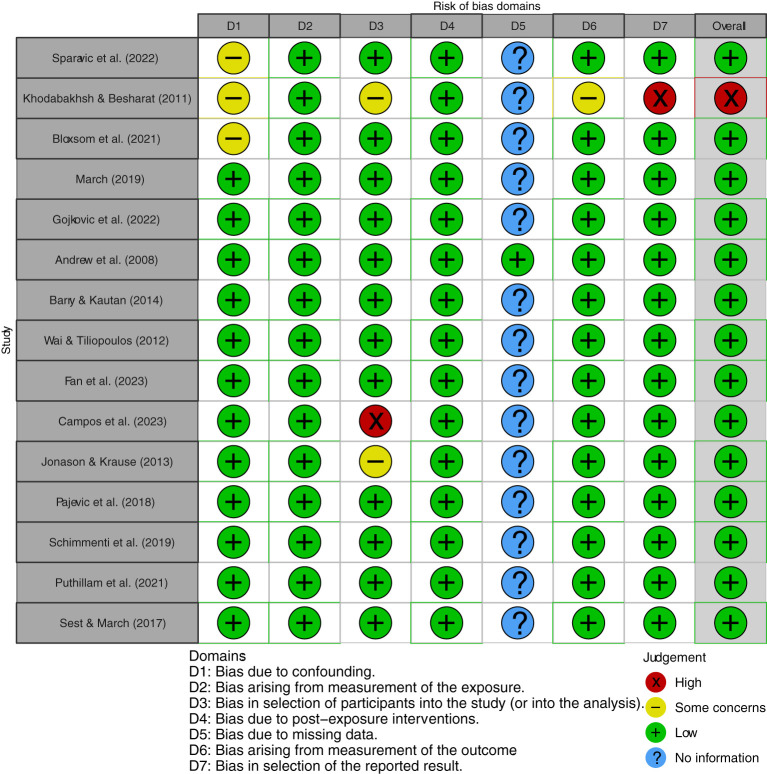
Traffic light plot used to assess the quality of the included studies.

**Figure 3:**
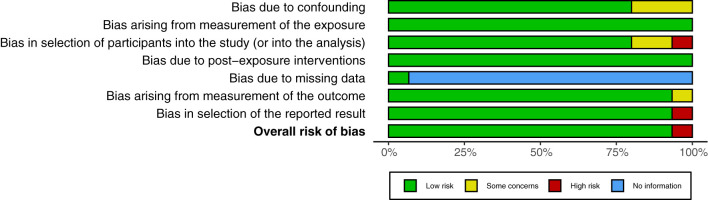
Summary plot used to assess the quality of the included studies.

**Figures 2** and **3**, respectively, depict the traffic light plot and the summary plot used to evaluate the quality of the 15 studies initially included (i.e., prior to quality evaluation; see **Figure 1**). The overall risk of bias was low, except for the study by Khodabakhsh and Besharat (84), which demonstrated a high risk of bias, particularly because of the results not being reported fully and due to some concerns about confounding, selection of study participants, and measurement of outcome. Thus, this study was not deemed to be of sufficient quality for inclusion in this systematic review and meta-analysis, and hence was dropped. All the included studies, except Andrew et al. (41), did not state anything about missing data. For the other six criteria for assessment of risk of bias, all the studies scored in the range of 80-100%. Finally, 14 studies were retained for this systematic review and meta-analysis.

### Characteristics of the included studies

This systematic review and meta-analysis included 14 studies (N = 5,328) from several different countries. Among these 14 studies, four were conducted on people from Australia, two included people from Serbia, one each included people from United Kingdom, England, Portugal, Italy, and China. Three studies included participants from multiple countries. March (49) recruited participants from North-west and South-east Europe, Oceania, USA, and South-east Asia. Puthillam et al. (59) included participants from 17 different nationalities such as India, USA, etc. in their study, while Sest and March (52) recruited participants from Australia, USA, and other countries. The sample sizes in these studies ranged from 139 to 799. With such wide variations in the representation of participants from these countries, the subgroup analysis pertaining to country of the participants was not carried out. Intriguingly, all the included studies followed a cross-sectional research design. The detailed characteristics of the selected studies have been presented in **Table 1**.

**Table 1:** Summary characteristics of studies.

S. No.	Authors & year of publication	Study design	Sample size and location	Measures of Dark Triad	Measures of empathy	Outcomes of the study	Correlations of AE with Dark Triad traits	Correlations of CE with Dark Triad traits
1.	Bloxsom et al. (2021)(14)	Cross-sectional design	262 females, United Kingdom	SD3	QCAE	Affective & cognitive empathy correlated significantly negatively with all the three Dark Triad traits. However, cognitive empathy did not correlate significantly with Narcissism.	AE & Narcissism (r = -.153), AE & Machiavellianism (r = -.144), AE & Psychopathy (r= -.221)	CE & Narcissism (r = .058 NS), CE & Machiavellianism (r = -.161), CE & Psychopathy (r=-.310)
2.	March (2019)(49)	Correlational design	733 (517 females, 216 males), North-west Europe, Oceania, USA, South-east Asia, South-east Europe	LSRP	EQ	Affective & cognitive empathy correlated significantly negatively with Psychopathy.	AE & Psychopathy (r = -.12)	CE & Psychopathy (r = -.57)
3.	Gojković et al. (2022)(85)	Cross-sectional design	263 (155 female, 108 male) school and university students, Serbia	SD3	ACME	CE had significant positive correlations with Machiavellianism and Narcissism.	AE & Narcissism (-.18), AE & Machiavellianism (-.29), AE & Psychopathy (-.52)	CE & Narcissism (.24), CE & Machiavellianism (.13), CE & Psychopathy (-.05, NS)
4.	Barry et al. (2014)(86)	Cross-sectional design	185 (26 female, 159 male) adolescents, USA	Narcissistic Personality Inventory for Children	TEQ	AE and Narcissism correlated significantly negatively.	AE & Narcissism (-.16)	---
5.	Wai and Tiliopoulos (2012)(32)	Cross-sectional design	139 (106 females, 33 males) university students, Australia	Mach-IV (Machiavellianism), NPI (Narcissism), LSRP (Psychopathy)	EQ	AE & CE correlated negatively and significantly with all the Dark Triad traits, while CE correlated significantly but positively with Narcissism.	AE & Narcissism (-.21), AE & Machiavellianism (-.40), AE & Psychopathy (-.52)	CE & Narcissism (.18), CE & Machiavellianism (-.08, NS), CE & Psychopathy (-.16, NS)
6.	Campos et al. (2023)(40)	Cross-sectional design	515 (307 female, 208 male) community sample, Portugal	LSRP	QCAE	Both AE & CE correlated significantly negatively with Psychopathy.	AE & Psychopathy (-.279)	CE & Psychopathy (-.234)
7.	Jonason and Krause (2013)(35)	Cross-sectional design	320 (242 female, 78 male), Australia	DTDD	BES	Both AE & CE correlated significantly negatively with all the Dark Triad traits, except for the non-significant correlation of AE with Narcissism.	AE & Narcissism (-.00, NS), AE & Machiavellianism (-.21), AE & Psychopathy (-.38)	CE & Narcissism (-.14), CE & Machiavellianism (-.19), CE & Psychopathy (-.23)
8.	Pajevic et al. (2018)(42)	Cross-sectional design	576 (326 female, 250 male), Serbia	Self-report Psychopathy Scale-Short form (Psychopathy), Mach-IV (Machiavellianism), NPI (Narcissism)	BES	AE & CE correlated negatively and significantly with all the Dark Triad traits, while CE correlated significantly but positively with Narcissism.	AE & Narcissism (-.20), AE & Machiavellianism (-.27), AE & Psychopathy (-.34)	CE & Narcissism (.17), CE & Machiavellianism (-.16), CE & Psychopathy (-.14)
9.	Schimmenti et al. (2019)(48)	Cross-sectional design	799 (439 female, 360 male), Italy	DTDD	EQ	AE & CE correlated negatively and significantly with all the Dark Triad traits, while CE did not correlate significantly with Narcissism.	AE & Narcissism (-.21), AE & Machiavellianism (-.30), AE & Psychopathy (-.41)	CE & Narcissism (-.06, NS), CE & Machiavellianism (-.09), CE & Psychopathy (-.18)
10.	Puthillam et al. (2021)(59)	Cross-sectional design	212 (150 female, 62 male), 17 nationalities including India and USA	SD3	BES	AE & CE correlated negatively and significantly with all the Dark Triad traits, except Narcissism.	AE & Narcissism (-.07, NS), AE & Machiavellianism (-.30), AE & Psychopathy (-.25)	CE & Narcissism (-.03, NS), CE & Machiavellianism (-.15), CE & Psychopathy (-.38)
11.	Sest and March (2017)(52)	Cross-sectional design	415 (260 female, 150 male, 5 others), Australia, USA, and other nationalities	Psychopathy subscale of Short Dark Triad	EQ	AE (but not CE) was significantly correlated with Psychopathy.	AE & Psychopathy (-.35)	CE & Psychopathy (-.04, NS)
12.	Sparavec et al. (2022)(54)	Cross-sectional design	239 (131 female, 104 male, 4 others), Australia	SD3	EQ-Short version	AE correlated significantly negatively with Machiavellianism and Psychopathy, while CE did not correlate significantly with any Dark Triad trait.	AE & Narcissism (.02, NS), AE & Machiavellianism (-.26), AE & Psychopathy (-.26)	CE & Narcissism (.09, NS), CE & Machiavellianism (-.10, NS), CE & Psychopathy (-.09, NS)
13.	Fan et al. (2023)(43)	Cross-sectional design	420 (287 female, 133 male), China	DTDD	IRI- Chinese version	AE correlated significantly negatively with Machiavellianism.	AE & Machiavellianism (-.29)	CE & Machiavellianism (-.01, NS)
14.	Andrew et al. (2008)(41)	Cross-sectional design	250 (150 female, 150 male), England	Mach-IV	EQ-Short version	AE (but not CE) correlated significantly with Machiavellianism.	AE & Machiavellianism (-.404)	CE & Machiavellianism (-.056, NS)

NS, Not significant.

AE, Affective Empathy; CE, Cognitive Empathy; QCAE, Questionnaire of Cognitive & Affective Empathy; SD3, Short Dark Triad of Personality; LSRP, Levenson Self-report Psychopathy Scale (LSRP); EQ, Empathy Quotient; ACME, Affective and Cognitive Measure of Empathy; TEQ, Toronto Empathy Questionnaire; NPI, Narcissistic Personality Inventory; DTDD, Dark Triad Dirty Dozen; BES, Basic Empathy Scale; IRI, Interpersonal Reactivity Index.

### Cognitive empathy and the dark triad

**Figure 4:**
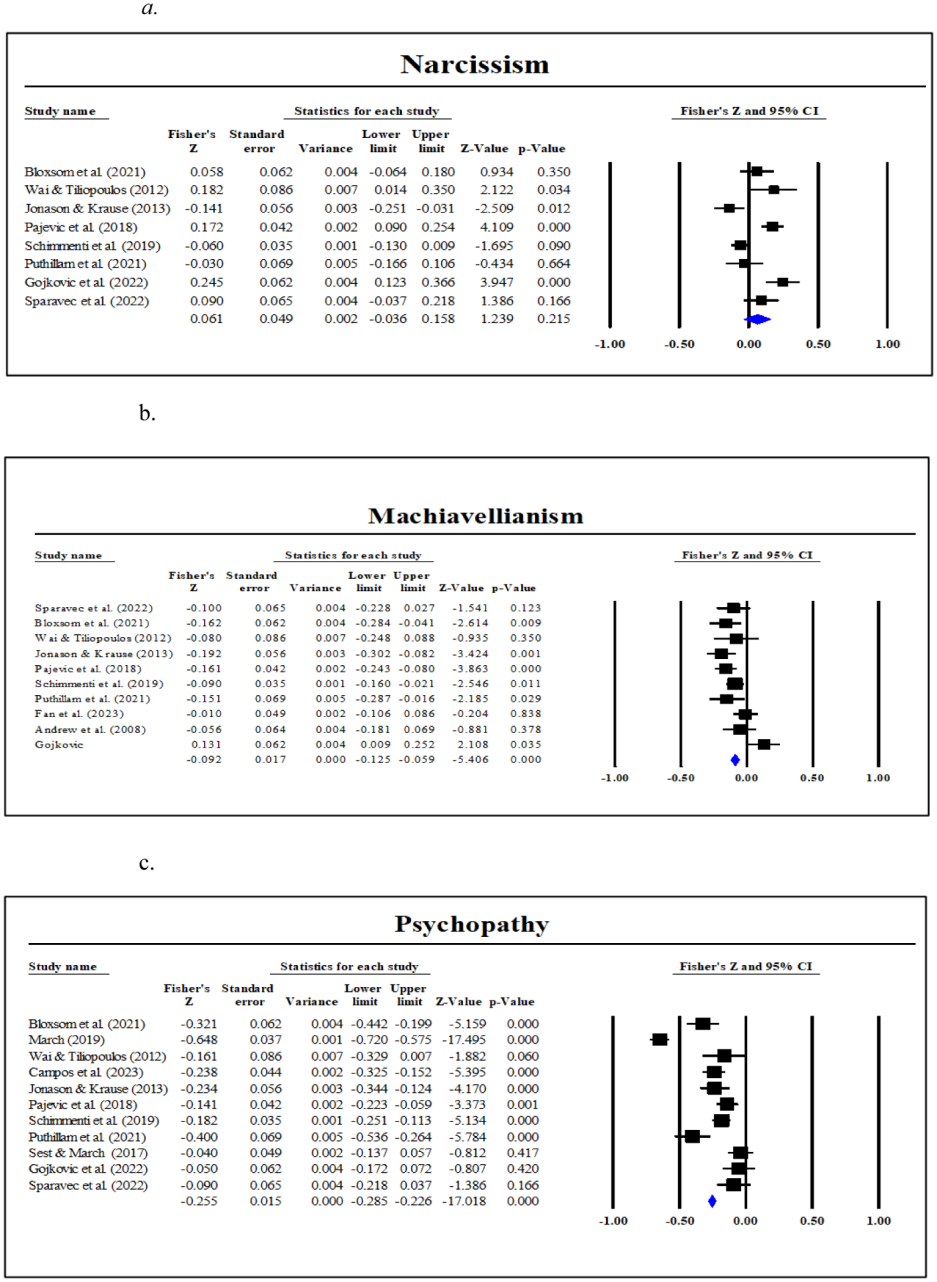
Meta-correlation of cognitive empathy with **(A)** Narcissism, **(B)** Machiavellianism, and **(C)** Psychopathy.

Thirteen studies analyzed the relationship between cognitive empathy and the Dark Triad traits, where eight studies explored the correlation of cognitive empathy with Narcissism, while 10 and 11 studies explored the association of cognitive empathy with Machiavellianism and Psychopathy, respectively. The analyzed Fisher r-to-z-transformed correlation coefficients for Narcissism ranged from -.030 to .245, with most of the values (62.50%) indicating a positive association across studies. The estimated Fisher r-to-z-transformed correlation coefficient, based on the random-effects model, was r = .061 (95% CI: -.036 to.159), as depicted in **Figure 4A**. The Fisher r-to-z-transformed correlation coefficients analyzed for Machiavellianism ranged from -.010 to -.192, with all values suggesting a negative association across studies. Using a random-effects model, the estimated Fisher r-to-z-transformed correlation coefficient was r = -.089 (95% CI: -.145 to -.032), as shown in **Figure 4B**. Similarly, for Psychopathy, Fisher r-to-z-transformed correlation coefficients ranged from -.121 to -.576, with all the values indicating a negative correlation across studies. The estimated Fisher r-to-z-transformed correlation coefficient, based on the random-effects model, was r = -.229 (95% CI: -.352 to -.106) (see **Figure 4C**).

The meta-analytic results for the association between cognitive empathy and Narcissism have been reported for the random-effects model as the Q-test highlighted considerable differences among the study outcomes (Q (7) = 43.571, *p* <.001), with an I² value of 83.93%, H statistic of 2.49, and τ² of 0.016, pointing to substantial heterogeneity in findings across studies. Such high heterogeneity suggests that the differences among studies may not be solely due to random variation. The random-effects model, which accounts for this variability, yielded a non-significant association between cognitive empathy and Narcissism, as evidenced by a z-score of 1.239 (*p* = .215). Based on the random-effects model, the calculated prediction interval was -0.174 to 0.296. The wide prediction interval further indicates that the association between cognitive empathy and Narcissism was not consistently positive or significant across different contexts. **Table 2** shows a summary of the values.

**Table 2:** Random-effects meta-analysis model of cognitive empathy for Narcissism, Machiavellianism, and Psychopathy .

Dark Triad traits	Pooled results [95% CI]	Prediction intervals [95% CI]	Heterogeneity							Publication Bias	
			τ^2^	τ	I^2^	H	Q	df	p	Egger’s Test	Rank Test
Narcissism	0.061 [-0.036, 0.158]	[-0.174, 0.296]	0.016	0.126	83.93%	2.49	43.57	7	.000	2.447	0.035
Machiavelli-anism	-0.089 [-0.145, -0.032]	[-0.239, 0.061]	0.005	0.071	62.54%	1.63	24.02	9	.004	0.732	0.045
Psychopathy	-0.229 [-0.352, -0.106]	[-0.640, -0.182]	0.040	0.2	94.03%	4.09	167.52	10	.000	4.357	-0.072

CI, Class Interval.

A subgroup analysis was conducted to examine whether narcissism measurement scales (NPI, SD3, DTDD) and empathy scales (EQ, BES, TEQ, QCAE) moderated the relationship between narcissism and cognitive empathy. The overall correlation was .40 (95% CI: .002 to .079, *p* = .041), suggesting a weak but statistically significant positive relationship between narcissism and cognitive empathy. However, heterogeneity was substantially high (Q = 43.034, df = 6, *p* <.001, I² = 86.06%), indicating significant variability in effect sizes across studies. Subgroup analyses based on narcissism measurement scales and empathy scales did not significantly reduce heterogeneity, suggesting that differences in measurement tools do not account for the variation observed.

With respect to Machiavellianism, heterogeneity analysis produced a Q-value of 24.03 (df = 9, *p* = .004) with an I² of 62.54% and H statistic of 1.63, indicating moderate heterogeneity. Tau-squared (τ²) was estimated at 0.005, with a standard error of 0.004, suggesting some variability in effect sizes across studies, although not as high as in Narcissism. The prediction interval for the pooled effect size ranged from -0.239 to 0.061 (see **Table 2**). This interval indicates that, while the overall estimated effect size suggests a negative association, the range within which future studies’ true effect sizes may fall includes both negative and values close to zero. This variability highlights that the relationship may differ across contexts or study-specific factors, suggesting limited generalizability of the observed association. As such, while the pooled effect suggests an overall trend, individual studies may observe weaker or non-significant associations.

A subgroup analysis was conducted to examine whether Machiavellianism measurement scales (MACH-IV, SD3, DTDD) and cognitive empathy scales (EQ, BES, TEQ, QCAE) moderated the relationship between Machiavellianism and cognitive empathy. The overall correlation was -.091 (95% CI: -.125 to -.057, *p* <.001), suggesting a small but significant negative relationship between Machiavellianism and cognitive empathy. However, heterogeneity was moderate-to-high (Q = 24.008, df = 8, *p* = .002, I² = 66.68%), indicating significant variability in effect sizes across studies. Subgroup analyses based on Machiavellianism measurement scales and cognitive empathy scales did not significantly reduce heterogeneity, suggesting that differences in measurement tools do not account for the variation observed.

For Psychopathy, considerably high heterogeneity was noted across studies (Q (10) = 167.523, *p*<.0001, H statistic of 4.09, and an I² statistic of 94.03%), demonstrating substantial variability among the studies. Additionally, the τ² estimate was 0.040, further supporting the presence of significant between-study variability, which challenges the assumption of homogeneity inherent in the fixed-effects model. The random-effects model, which accounts for between-study variance, yielded a pooled effect size of -0.229, with 95% CI ranging between -0.352 to -0.106. This result was statistically significant (z= -3.65, *p*<.0001), suggesting a consistent negative association across studies. The wider confidence interval in the random-effects model reflects the high degree of heterogeneity. The prediction interval was -0.640 to 0.182. This interval suggests that while the overall effect size from the random-effects model is negative (-0.229), future studies could observe either negative or positive effect sizes within this range. **Table 2** summarizes these values.

A subgroup analysis was conducted to examine whether psychopathy measurement scales (SRP-SF, LSRP, DTDD, SD3) and cognitive empathy scales (EQ, BES, TEQ, QCAE) moderated the relationship between psychopathy and cognitive empathy. The overall correlation was -.259 (95% CI: -.287 to -.230, *p* <.001), suggesting a moderate and significant negative relationship between psychopathy and cognitive empathy. However, heterogeneity was very high (Q = 160.724, df = 9, *p* <.001, I² = 94.40%), indicating substantial variability in effect sizes across studies. Subgroup analyses based on psychopathy measurement scales and cognitive empathy scales did not significantly reduce heterogeneity, suggesting that differences in measurement tools do not account for the variation observed.

**Figure 5:**
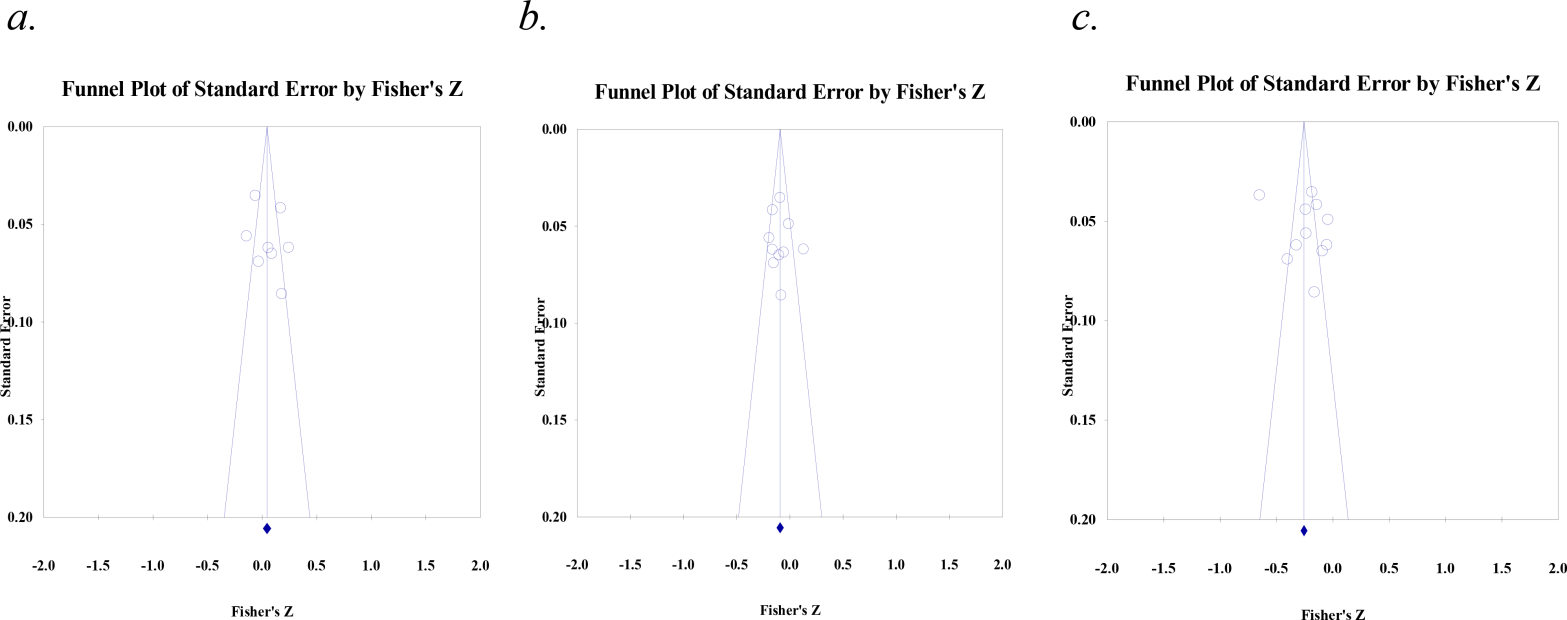
Funnel plots for **(A)** Narcissism, **(B)** Machiavellianism, and **(C)** Psychopathy.

Egger’s test for funnel plot asymmetry for Narcissism indicated an intercept of 2.44 (95% CI: -5.761, 10.655), with a p-value of .49. This non-significant p-value (*p* >.05) suggests the absence of asymmetry in the funnel plot (see **Figure 5A**), which may indicate no significant publication bias among the included studies. The same result (no potential publication bias) was noted for Machiavellianism (intercept= 0.73, 95% CI: -4.26, 5.73, *p*= .74) and Psychopathy (intercept= 4.36, 95% CI: -6.62, 15.34, *p*= .39) (see **Figures 5B, C**). Providing further support to these results, the Begg and Mazumdar rank correlation test (with continuity correction) indicated no significant publication bias for Narcissism (Kendall’s tau = 0.035, z = 0.123, *p*= .90), Machiavellianism (Kendall’s tau = 0.045, z = 0.179, *p*= .85), or Psychopathy (Kendall’s tau = -0.090, z = 0.389, *p*= .69).

### Affective empathy and the dark triad

**Figure 6:**
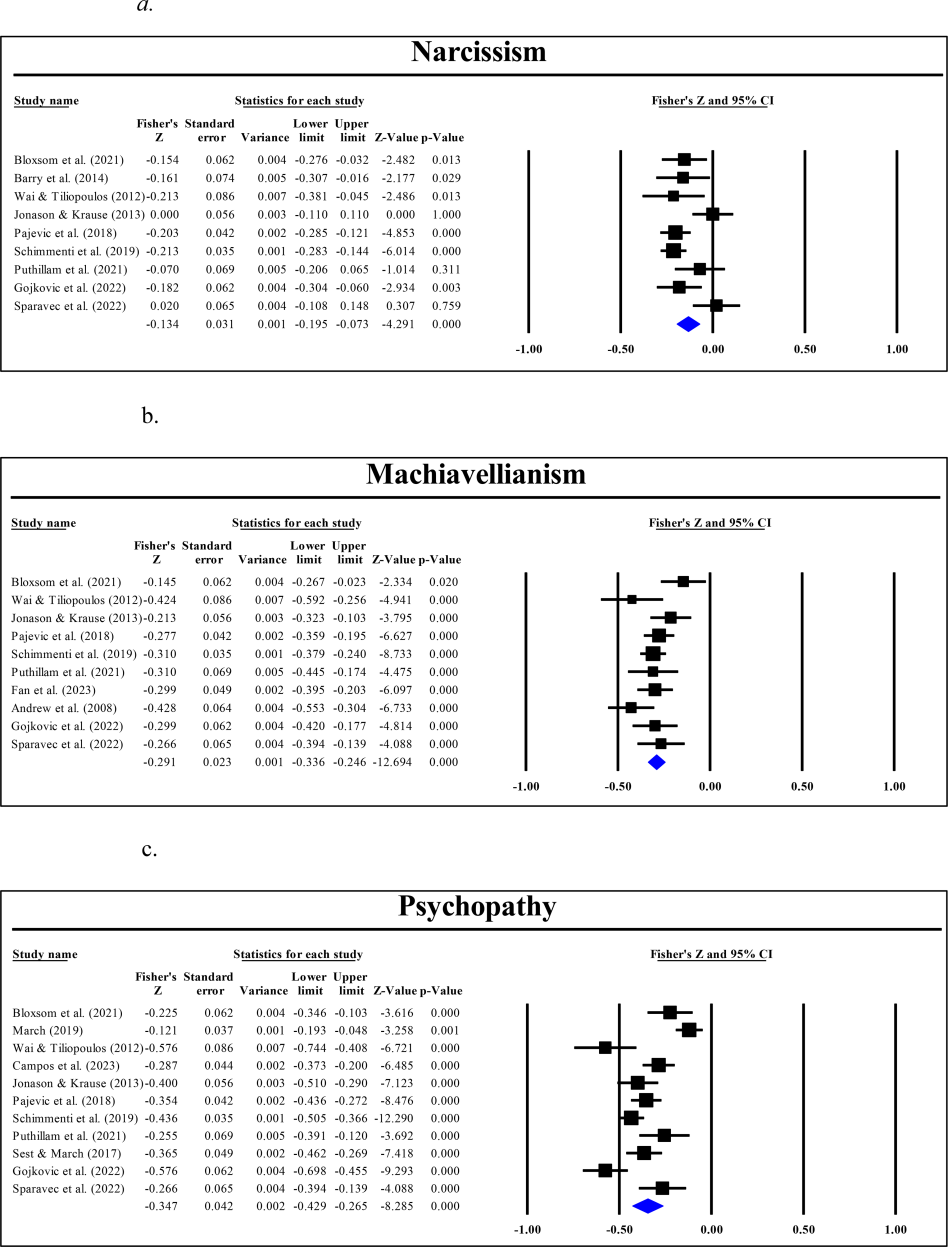
Meta-correlation of affective empathy with **(A)** Narcissism, **(B)** Machiavellianism, and **(C)** Psychopathy.

Nine studies analyzed the relationship between affective empathy and the Dark Triad trait of Narcissism, while 10 and 11 studies explored the association of affective empathy with Machiavellianism and Psychopathy, respectively. The analyzed Fisher r-to-z-transformed correlation coefficients for Narcissism ranged from .000 to -.213, with most of the values (77.78%) indicating a negative association across studies. The estimated Fisher r-to-z-transformed correlation coefficient, based on the random-effects model, was r = -.134 (95% CI: -.195 to -.073), as depicted in **Figure 6A**. The Fisher r-to-z-transformed correlation coefficients analyzed for Machiavellianism ranged from -.428 to -.145, with all values suggesting a negative association across studies. Using a random-effects model, the estimated Fisher r-to-z-transformed correlation coefficient was r = -.291 (95% CI: .324 to -.257), as shown in **Figure 6B**. Similarly, for Psychopathy, Fisher r-to-z-transformed correlation coefficients ranged from -.121 to -.576, with all the values indicating a negative correlation across studies. The estimated Fisher r-to-z-transformed correlation coefficient, based on the random-effects model, was r = -.347 (95% CI: -.429 to -.265) (see **Figure 6C**).

The overall findings indicate that the estimated effect size for Narcissism demonstrated significant variability, as evidenced by a z-score of -4.291 (*p*<.0001). The results of the Q-test highlight considerable differences among the study outcomes (Q (8) = 20.889, *p*<.001), with an H statistic of 1.63, a τ² of 0.005 and an I² value of 61.70%, pointing to substantial heterogeneity in findings across studies examining affective empathy in Narcissism. The prediction interval, ranging from -0.285 to 0.017, suggests that subsequent research findings are expected to fall within this range. Therefore, despite the presence of heterogeneity, the overall outcomes of the studies are likely to trend similarly to the average outcome observed here.

A meta-analytic subgroup analysis was conducted to examine whether the choice of narcissism and empathy scales contributed to heterogeneity in the relationship between narcissism and affective empathy. The overall correlation remained -.162 (95% CI: -.198 to -.125, *p* <.001), with moderate heterogeneity (Q = 13.59, df = 7, *p* = .059, I² = 48.49%). Subgroup analyses based on narcissism measurement scales (NPI, SD3, DTDD) and empathy scales (EQ, BES, TEQ) did not significantly reduce heterogeneity, suggesting that the variability in effect sizes is not primarily driven by differences in measurement tools.

With respect to Machiavellianism, the estimated effect size varied significantly, with a z-score of -17.080 (*p*<.0001). The results of the Q-test demonstrated homogeneity among the outcomes of the included studies (Q (9) = 15.142, *p*= .087), indicating that any variations in effect sizes across the studies could be attributed to sampling error rather than true differences between the studies. A τ² value of 0.002 indicates minimal variability in the true effect sizes among the studies and the same can be inferred about the H statistic of 1.30. These reinforce the finding from the Q-test that the studies are fairly consistent in their outcomes. An I² statistic of 40.56% indicates moderate heterogeneity among the studies. While there is some variability in the effect sizes, it is not excessively high, suggesting that a considerable portion of the variation could still be due to random sampling error rather than true differences. Thus, as per the fixed-effects model, a 95% prediction interval was established, ranging from -0.385 to -0.197, suggesting that future research findings are likely to fall within this range. For Psychopathy, the estimated effect size varied significantly with a z-score of -8.285 (*p*<.0001). Considerable heterogeneity was noted across studies (Q (10) = 73.292, *p*<.0001), with an H statistic of 2.71, a τ² value of 0.016 and an I² statistic of 86.36%. The prediction interval ranged from -0.608 to -0.086. **Table 3** shows a summary of the values.

**Table 3:** Random-effects (for narcissism and psychopathy) and Fixed-effects (for Machiavellianism) meta-analysis model of affective empathy.

Dark Triad traits	Pooled results [95% CI]	Prediction intervals [95% CI]	Heterogeneity							Publication Bias	
			τ^2^	τ	I^2^	H	Q	df	p	Egger’s Test	Rank Test
**Narcissism**	-0.134 [-0.195, -0.073]	[-0.285, 0.017]	0.005	0.072	61.70%	1.62	20.89	8	.007	2.53	0.14
**Machiavelli-anism**	-0.291 [-0.324, -0.257]	[-0.385, -0.197]	0.002	0.045	40.56%	1.30	15.14	9	.087	-0.56	-0.09
**Psychopathy**	-0.347 [-0.429, -0.265]	[-0.608, -0.086]	0.016	0.127	86.36%	2.71	73.29	10	.000	-2.78	-0.04

A subgroup analysis was conducted to examine whether Machiavellianism measurement scales (Mach-IV, SD3, DTDD) and empathy scales (EQ, BES, TEQ, QCAE, IRI-C) moderated the relationship between Machiavellianism and affective empathy. The overall correlation was -.285 (95% CI: -.316 to -.252, *p* <.001), suggesting a moderate negative relationship between Machiavellianism and empathy. However, heterogeneity was moderate (Q = 14.988, df = 8, *p* = .059, I² = 46.62%), indicating some variability in effect sizes across studies. Subgroup analyses based on Machiavellianism measurement scales and empathy scales did not significantly reduce heterogeneity, suggesting that differences in measurement tools do not account for the variation observed. A subgroup analysis was conducted to examine whether psychopathy measurement scales (SRP-SF, LSRP, DTDD, SD3) and affective empathy scales (EQ, BES, TEQ, QCAE) moderated the relationship between psychopathy and affective empathy. The overall correlation was -.322 (95% CI: -.348 to -.294, p <.001), suggesting a moderate and significant negative relationship between psychopathy and affective empathy. However, heterogeneity was very high (Q = 72.280, df = 9, p <.001, I^2^ = 87.55%), indicating substantial variability in effect sizes across studies. Subgroup analyses based on psychopathy measurement scales and affective empathy scales did not significantly reduce heterogeneity, suggesting that differences in measurement tools do not account for the variation observed.

**Figure 7:**
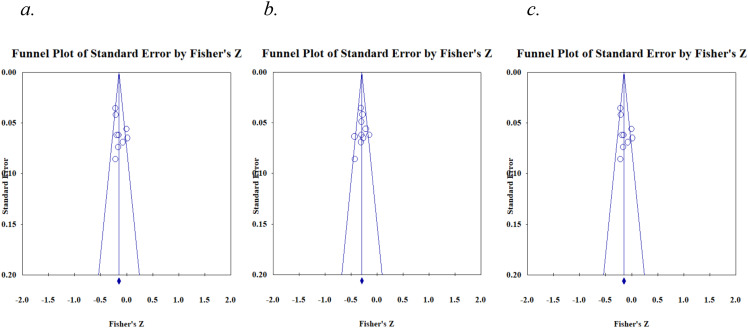
Funnel plots for **(A)** Narcissism, **(B)** Machiavellianism, and **(C)** Psychopathy.

Egger’s test for funnel plot asymmetry for Narcissism indicated an intercept of 2.53 (95% CI: -1.88, 6.94), with a p-value of .22. This non-significant p-value (*p* > .05) suggests the absence of asymmetry in the funnel plot (see **Figure 7A**), which may indicate no significant publication bias among the included studies. The same result (no potential publication bias) was noted for Machiavellianism (intercept= -0.56, 95% CI: -4.53, 3.40, *p*= .75) and Psychopathy (intercept= -2.78, 95% CI: -10.07, 4.51, *p*= .41) (see **Figures 7B, C**). Providing further support to these results, the Begg and Mazumdar rank correlation test (with continuity correction) indicated no significant publication bias for Narcissism (Kendall’s tau = 0.14, z = 0.52, *p*= .60), Machiavellianism (Kendall’s tau = -0.09, z = 0.36, *p*= .72), or Psychopathy (Kendall’s tau = -0.04, z = 0.16, *p*= .88).

## Discussion

The findings of this systematic review and meta-analysis offer novel insights into the nuanced relationships between the Dark Triad traits—Narcissism, Machiavellianism, and Psychopathy—and the two types of empathy: cognitive empathy (CE) and affective empathy (AE). Unlike previous studies, which often examined these traits in isolation or focused on limited empathy measures, this meta-analysis synthesizes findings across multiple studies to reveal distinct empathy profiles for each trait. Notably, the study provides meta-analytic evidence highlighting the variability of CE and AE deficits in Dark Triad across studies, as well as the robust and consistent AE deficits in Machiavellianism and psychopathy. Furthermore, the integration of prediction intervals adds a unique layer of analysis, offering insights into the range of expected outcomes in future research. By systematically exploring heterogeneity and prediction intervals, this study not only reaffirms established theories but also identifies critical gaps and variability in the empathy profiles of Dark Triad traits, offering a foundation for more nuanced theoretical models and practical applications.

Firstly, the relationship between narcissism and CE was nuanced but not statistically significant, with a weak positive association (r = .047) and high heterogeneity (I² = 83.93%). This variability aligns with findings from previous studies suggesting that narcissistic individuals, particularly those exhibiting grandiose narcissism, may retain or even enhance CE, leveraging it strategically in social contexts to maintain admiration and influence (29, 44). Conversely, vulnerable narcissism has been associated with broader empathy deficits (86), indicating that subtypes of narcissism may modulate the relationship between CE and narcissism. The wide prediction interval (PI: -0.122 to 0.216) further highlights contextual variability, suggesting that situational moderators, such as environmental demands or interpersonal goals, could shape narcissistic individuals’ engagement with cognitive empathy. These findings underscore the need for future research to disaggregate narcissistic subtypes and examine contextual factors that influence how narcissism interacts with CE. The observed high heterogeneity across studies is not attributable to variations in measures used to assess narcissism or CE as the substantial heterogeneity (I^2^ = 86.06%), obtained from subgroup analysis examining whether narcissism and empathy measurement scales moderated their association,suggests that other factors, beyond measurement scales, contribute to variability in effect sizes.

The association between AE and narcissism was more consistent, with a significant negative correlation (r = -.134, 95% CI: -.195 to -.073) indicating that narcissistic individuals tend to have reduced AE. This aligns with their self-centered and emotionally detached characteristics, which diminish their ability to emotionally resonate with others (87, 88). However, the prediction interval (PI: -0.285 to 0.017) suggests that in some contexts, this negative association may be minimal or even negligible, further emphasizing the complex and variable nature of narcissism’s impact on AE. The negative relationship between narcissism and affective empathy remains consistent regardless of the measurement scale used. While previous research [e.g., (89)] has highlighted theoretical differences between narcissism scales (e.g., NPI capturing grandiosity vs. DTDD capturing antagonistic traits), these methodological differences do not appear to substantially impact the observed effect sizes in this meta-analysis. The moderate heterogeneity (I² = 48.49%) indicates that other factors may be contributing to variation in effect sizes across studies. Potential sources of this unexplained heterogeneity may include sample characteristics (e.g., cultural differences, gender distributions) or study design variations.

Machiavellianism showed consistent negative associations with both CE and AE, particularly the latter (r = -.291). The strong and robust negative correlation between AE and Machiavellianism, with a narrow prediction interval (PI: -0.385 to -0.197) and low heterogeneity (I² = 40.56%), suggests that these individuals display enduring deficits in AE across contexts. This finding aligns with the manipulative, emotionally detached nature of Machiavellianism, where emotional resonance is largely absent (90). Interestingly, the moderate heterogeneity observed in the CE-Machiavellianism relationship indicates that CE may be retained to some extent, potentially aiding their strategic manipulation (43). This selective engagement with CE underscores how Machiavellians may cognitively understand others’ emotions while remaining emotionally detached, enabling them to exploit social interactions for personal gain. The moderate-to-high heterogeneity (I² = 66.68%) suggests that other factors, beyond measurement scales, contribute to variability in effect sizes.

While prior studies have discussed differences between Mach-IV (a traditional trait measure) and SD3 (a shorter measure focused on socially malevolent traits), our findings suggest that these theoretical differences do not significantly moderate the relationship of Machiavellianism with empathy, due to the observed moderate heterogeneity (I² = 46.62%). Similar to the findings for narcissism and affective empathy, the moderate heterogeneity suggests that cultural, demographic, and methodological variations across studies may be influencing the observed effect sizes. For example, individualistic cultures may show stronger negative relationships between Machiavellianism and empathy compared to collectivistic cultures. Additionally, study sample characteristics (e.g., student vs. general population) may introduce variability.

Psychopathy exhibited the strongest and most consistent empathy deficits, particularly in AE (r = -.347). This aligns with psychopathy’s well-documented characteristics, such as lack of remorse, shallow emotions, and general affective detachment (22, 24). However, the high heterogeneity in the CE-psychopathy relationship (I² = 94.03%) suggests that cognitive empathy may vary across psychopathy subtypes or situational contexts. For instance, primary psychopathy, associated with interpersonal manipulation, may retain CE, whereas secondary psychopathy, characterized by impulsivity, may show broader deficits (40). These findings support prior research indicating that psychopathy’s affective empathy deficits are central to its antisocial behaviors, but the role of CE in facilitating manipulation warrants further exploration. Variations in measurement scales also did not account for variability in effect sizes due to the very high heterogeneity (I² = 87.55%) in measurement scales. With respect to cognitive empathy too, the exceptionally high heterogeneity (I² = 94.40%) in measurement-wise subgroup analysis indicates that factors beyond the type of measurement scales are influencing the variation in effect sizes. Factors responsible for such heterogeneity may be gender or cultural factors. However, gender-wise subgroup analysis was not feasible in the present study.

Interestingly, these findings contribute to the ongoing debate about empathy’s adaptive role in socially aversive personalities. While AE deficits are pervasive across all three traits, CE appears to be selectively retained or strategically deployed, particularly in narcissism and Machiavellianism. Emerging constructs such as “Dark Empaths” (29) further complicate this picture, suggesting that individuals with high levels of CE and AE may combine empathy with manipulative tendencies, leading to fewer negative outcomes compared to traditional Dark Triad profiles. These insights challenge the assumption of universal empathy deficits across the Dark Triad and underscore the importance of studying individual and situational factors that shape empathy in these traits.

The methodological variability in existing studies also warrants attention. Differences in measurement tools, such as the Basic Empathy Scale versus the Interpersonal Reactivity Index, may contribute to inconsistencies in reported findings (40), though our findings failed to support this. Additionally, cultural norms, gender differences, and study designs likely influence the expression of Dark Triad traits and their association with empathy (43). These factors highlight the need for future studies to employ standardized methods and consider contextual moderators to enhance the comparability and generalizability of findings.

This research, while comprehensive in synthesizing studies on empathy within Dark Triad traits, has several limitations. Firstly, the meta-analysis included only cross-sectional studies, limiting causal interpretations. Additionally, publication and language biases may persist despite efforts to mitigate them. The moderate to high heterogeneity observed, particularly in CE findings for narcissism and psychopathy, reflects variability in study methodologies and populations, further limiting generalizability. Moreover, reliance on self-report measures raises concerns about social desirability and self-awareness biases, particularly in traits characterized by manipulation and self-focus. Additionally, while we conducted subgroup analyses based on measurement scales for both Dark Triad traits and empathy, subgroup analyses by gender and country were not feasible due to insufficient or incomplete data in several studies. Many studies did not report separate effect sizes for male and female participants or provide country-specific breakdowns. As a result, potential cultural or gender-based moderating effects on the relationship between Dark Triad traits and empathy remain unexplored. Future meta-analytic research should prioritize including studies that report gender-stratified and country-specific effect sizes, allowing for a more nuanced understanding of these relationships across diverse populations.

Finally, the implications for both research and practice are notable. Understanding the empathy profiles of Dark Triad traits can enhance theoretical models and inform interventions aimed at mitigating antisocial behaviors. For example, organizations may benefit from empathy training programs tailored to specific empathy deficits identified in individuals with these traits, fostering better interpersonal dynamics and reducing the adverse impacts of the Dark Triad on teamwork and leadership. Future research should prioritize longitudinal studies and examine emerging constructs such as “Dark Empaths” to further refine our understanding of empathy within the Dark Triad.

## Conclusion

In conclusion, this systematic review and meta-analysis underscores the complex role of empathy in Dark Triad personalities, where affective empathy is predominantly impaired, while cognitive empathy varies across traits. These findings hold critical practical implications. For physicians, pronounced affective empathy deficits in psychopathy can be addressed through emotion recognition training to enhance social functioning, while preserved cognitive empathy in narcissistic individuals can be leveraged in cognitive-behavioral therapy to foster prosocial behavior. In the case of Machiavellianism, comprehensive empathy-building programs targeting both cognitive and affective empathy deficits are crucial to mitigate manipulative behaviors. For mental health professionals, these insights can guide the development of diagnostic tools and tailored interventions for empathy deficits linked to antisocial tendencies, alongside empathy training programs to reduce manipulation and aggression, improving interpersonal outcomes. Beyond individual interventions, these findings provide a foundation for enhancing organizational dynamics through empathy-focused leadership training. Future research should explore empathy profiles across subtypes and situational factors to further refine interventions and advance our understanding of empathy in socially aversive personalities.

## Data Availability

The original contributions presented in the study are included in the article. Further inquiries can be directed to the corresponding author.

## References

[1] Shamay-Tsoory SG Aharon-Peretz J Perry D . Two systems for empathy: a double dissociation between emotional and cognitive empathy in inferior frontal gyrus versus ventromedial prefrontal lesions. Brain. (2009) 132:617–27. doi: 10.1093/brain/awn279 18971202

[2] Wispé L . The distinction between sympathy and empathy: To call forth a concept, a word is needed. J Pers Soc Psychol. (1986) 50:314–21. doi: 10.1037/0022-3514.50.2.314

[3] Cohen D Strayer J . Empathy in conduct-disordered and comparison youth. Dev Psychol. (1996) 32:988–98. doi: 10.1037/0012-1649.32.6.988

[4] Jolliffe D Farrington DP . Empathy and offending: A systematic review and meta-analysis. Aggression violent Behav. (2004) 9:441–76. doi: 10.1016/j.avb.2003.03.001

[5] Martingano AJ . *A dual process model of empathy* . New York, United States: The New School (2020).

[6] Yu CL Chou TL . A dual route model of empathy: A neurobiological prospective. Front Psychol. (2018) 9:2212. doi: 10.3389/fpsyg.2018.02212 30483202 PMC6243070

[7] Yan Z Hong S Liu F Su Y . A meta-analysis of the relationship between empathy and executive function. PsyCh J. (2020) 9(1):34–43. doi: 10.1002/pchj.3112 31394592

[8] Dimberg U Thunberg M Elmehed K . Unconscious facial reactions to emotional facial expressions. psychol Sci. (2000) 11:86–9. doi: 10.1111/1467-9280.00221 11228851

[9] Morelli SA Lieberman MD . The role of automaticity and attention in neural processes underlying empathy for happiness, sadness, and anxiety. Front Hum Neurosci. (2013) 7:160. doi: 10.3389/fnhum.2013.00160 23658538 PMC3647144

[10] Rameson LT Morelli SA Lieberman MD . The neural correlates of empathy: Experience, automaticity, and prosocial behavior. J Cogn Neurosci. (2012) 24:235–45. doi: 10.1162/jocn_a_00130 21878057

[11] Lockwood PL Seara-Cardoso A Viding E . Emotion regulation moderates the association between empathy and prosocial behavior. PloS One. (2014) 9:e96555. doi: 10.1371/journal.pone.0096555 24810604 PMC4014517

[12] Batson CD . These things called empathy: Eight related but distinct phenomena. In: Decety J Ickes W , editors. The social neuroscience of empathy. Cambridge: MIT Press (2009). p. 3–15. doi: 10.7551/mitpress/9780262012973.003.0002

[13] Hall JA Schwartz R . Empathy present and future. J Soc Psychol. (2019) 159:225–43. doi: 10.1080/00224545.2018.1477442 29781776

[14] Bloxsom CA Firth J Kibowski F Egan V Sumich AL Heym N . Dark shadow of the self: How the dark triad and empathy impact parental and intimate adult attachment relationships in women. Forensic Sci International: Mind Law. (2021) 2:100045. doi: 10.1016/j.fsiml.2021.100045

[15] Martingano AJ Konrath S . How cognitive and emotional empathy relate to rational thinking: empirical evidence and meta-analysis. J Soc Psychol. (2022) 162:143–60. doi: 10.1080/00224545.2021.1985415 35083952

[16] Davis MH . Measuring individual differences in empathy: Evidence for a multidimensional approach. J Pers Soc Psychol. (1983) 44:113–26. doi: 10.1037/0022-3514.44.1.113

[17] Eisenberg N Fabes RA Miller PA Fultz J Shell R Mathy RM . Relation of sympathy and personal distress to prosocial behavior: A multimethod study. J Pers Soc Psychol. (1989) 57:55–66. doi: 10.1037/0022-3514.57.1.55 2754604

[18] Murphy BA Lilienfeld SO . Are self-report cognitive empathy ratings valid proxies for cognitive empathy ability? Negligible meta-analytic relations with behavioral task performance. psychol Assess. (2019) 31:1062. doi: 10.1037/pas0000732 31120296

[19] Austin EJ Farrelly D Black C Moore H . Emotional intelligence, Machiavellianism and emotional manipulation: Does EI have a dark side? Pers Individ Dif. (2007) 43:179–89. doi: 10.1016/j.paid.2006.11.019

[20] Bacon AM Regan L . Manipulative relational behavior and delinquency: Sex differences and links with emotional intelligence. J Forensic Psychiatry Psychol. (2016) 27:331–48. doi: 10.1080/14789949.2015.1134625

[21] Barlow A Qualter P Stylianou M . Relationships between Machiavellianism, emotional intelligence and theory of mind in children. Pers Individ Dif. (2010) 48:78–82. doi: 10.1016/j.paid.2009.08.021

[22] Blair J Sellars C Strickland I Clark F Williams A Smith M . Theory of mind in the psychopath. J Forensic Psychiatry. (1996) 7:15–25. doi: 10.1080/09585189608409914

[23] Dadds MR Hawes DJ Frost AD Vassallo S Bunn P Hunter K . Learning to ‘talk the talk’: The relationship of psychopathic traits to deficits in empathy across childhood. J Child Psychol Psychiatry. (2009) 50:599–606. doi: 10.1111/j.1469-7610.2008.02058.x 19445007

[24] Dolan M Fullam R . Theory of mind and mentalizing ability in antisocial personality disorders with and without psychopathy. psychol Med. (2004) 34:1093–102. doi: 10.1017/S0033291704002028 15554579

[25] Richell RA Mitchell DG Newman C Leonard A Baron-Cohen S Blair RJR . Theory of mind and psychopathy: can psychopathic individuals read the ‘language of the eyes’? Neuropsychologia. (2003) 41:523–6. doi: 10.1016/S0028-3932(02)00175-6 12559146

[26] Shamay-Tsoory SG Harari H Aharon-Peretz J Levkovitz Y . The role of the orbitofrontal cortex in affective theory of mind deficits in criminal offenders with psychopathic tendencies. Cortex. (2010) 46:668–77. doi: 10.1016/j.cortex.2009.04.008 19501818

[27] Burghart M Mier D . No feelings for me, no feelings for you: A meta-analysis on alexithymia and empathy in psychopathy. Pers Individ Dif. (2022) 194:111658. doi: 10.1016/j.paid.2022.111658

[28] Paulhus DL Williams KM . The dark triad of personality: Narcissism, Machiavellianism, and psychopathy. J Res Pers. (2002) 36:556–63. doi: 10.1016/S0092-6566(02)00505-6

[29] Heym N Kibowski F Bloxsom CA Blanchard A Harper A Wallace L . The Dark Empath: Characterising dark traits in the presence of empathy. Pers Individ Dif. (2021) 169:110172. doi: 10.1016/j.paid.2020.110172

[30] Vonk J Zeigler-Hill V Mayhew P Mercer S . Mirror, mirror on the wall, which form of narcissist knows self and others best of all? Pers Individ Dif. (2013) 54:396–401. doi: 10.1016/j.paid.2012.10.010

[31] Heym N Firth J Kibowski F Sumich A Egan V Bloxsom CA . Empathy at the heart of darkness: Empathy deficits that bind the dark triad and those that mediate indirect relational aggression. Front Psychiatry. (2019) 10:95. doi: 10.3389/fpsyt.2019.00095 30930800 PMC6423894

[32] Wai M Tiliopoulos N . The affective and cognitive empathic nature of the dark triad of personality. Pers Individ Dif. (2012) 52:794–9. doi: 10.1016/j.paid.2012.01.008

[33] Jakobwitz S Egan V . The dark triad and normal personality traits. Pers Individ Dif. (2006) 40:331–9. doi: 10.1016/j.paid.2005.07.006

[34] Christie R Geis FL . Chapter I-why machiavelli. In: Studies in machiavellianism (1970). p. 1–9.

[35] Jonason PK Krause L . The emotional deficits associated with the Dark Triad traits: Cognitive empathy, affective empathy, and alexithymia. Pers Individ Dif. (2013) 55:532–7. doi: 10.1016/j.paid.2013.04.027

[36] Jones DN Paulhus DL . Introducing the short dark triad (SD3) a brief measure of dark personality traits. Assessment. (2014) 21:28–41. doi: 10.1177/1073191113514105 24322012

[37] Watson PJ Grisham SO Trotter MV Biderman MD . Narcissism and empathy: Validity evidence for the Narcissistic Personality Inventory. J Pers Assess. (1984) 48:301–5. doi: 10.1207/s15327752jpa4803_12 16367529

[38] Cleckley H . The mask of sanity: an attempt to clarify some issues about the so-called psychopathic personality. (5th ed.). Missouri, USA: Mosby (1976)

[39] Hoppenbrouwers SS Bulten BH Brazill A . Parsing fear: A reassessment of the evidence for fear deficits in psychopathy. Psychol Bullet. (2016) 142(6):573–600. doi: 10.1037/bul0000040 26854867

[40] Campos C Rocha NB Barbosa F . Dissociating cognitive and affective empathy across psychopathy dimensions: The role of interoception and alexithymia. Front Psychol. (2023) 14:1082965. doi: 10.3389/fpsyg.2023.1082965 37457066 PMC10345207

[41] Andrew J Cooke M Muncer SJ . The relationship between empathy and Machiavellianism: An alternative to empathizing–systemizing theory. Pers Individ Dif. (2008) 44:1203–11. doi: 10.1016/j.paid.2007.11.014

[42] Pajevic M Vukosavljevic-Gvozden T Stevanovic N Neumann CS . The relationship between the Dark Tetrad and a two-dimensional view of empathy. Pers Individ Dif. (2018) 123:125–30. doi: 10.1016/j.paid.2017.11.009

[43] Fan Q Wang X Liu Y . Can Machiavellianism not be prosocial? Roles of empathy and death anxiety. psychol Rep. (2023), 00332941231169665. doi: 10.1177/00332941231169665 37127436

[44] Schimmenti A Jonason PK Passanisi A La Marca L Di Dio N Gervasi AM . Exploring the dark side of personality: Emotional awareness, empathy, and the Dark Triad traits in an Italian sample. Current Psychol. (2017) 38:100–9. doi: 10.1007/s12144-017-9588-6

[45] Egger M Smith GD Schneider M Minder C . Bias in meta-analysis detected by a simple, graphical test. BMJ. (1997) 315:629–34. doi: 10.1136/bmj.315.7109.629 PMC2127453 9310563

[46] Shi L Lin L . The trim-and-fill method for publication bias: practical guidelines and recommendations based on a large database of meta-analyses. Medicine. (2019) 98:e15987. doi: 10.1097/MD.0000000000015987 31169736 PMC6571372

[47] McGuinness LA Higgins JP . Risk-of-bias VISualization (robvis): an R package and Shiny web app for visualizing risk-of-bias assessments. Res synthesis Methods. (2021) 12:55–61. doi: 10.1002/jrsm.1411 32336025

[48] Schimmenti A Jonason PK Passanisi A La Marca L Di Dio N Gervasi AM . Exploring the dark side of personality: Emotional awareness, empathy, and the Dark Triad traits in an Italian Sample. Curr Psychol. (2019) 38:100–9. doi: 10.1007/s12144-017-9588-6

[49] March E . Psychopathy, sadism, empathy, and the motivation to cause harm: New evidence confirms malevolent nature of the Internet Troll. Pers Individ Dif. (2019) 141:133–7. doi: 10.1016/j.paid.2019.01.001

[50] Lawrence EJ Shaw P Baker D Baron-Cohen S David AS . Measuring empathy: reliability and validity of the Empathy Quotient. psychol Med. (2004) 34:911–20. doi: 10.1017/S0033291703001624 15500311

[51] Preti A Vellante M Baron-Cohen S Zucca G Petretto DR Masala C . The Empathy Quotient: A cross-cultural comparison of the Italian version. Cogn neuropsychiatry. (2011) 16:50–70. doi: 10.1080/13546801003790982 20737328

[52] Sest N March E . Constructing the cyber-troll: Psychopathy, sadism, and empathy. Pers Individ Dif. (2017) 119:69–72. doi: 10.1016/j.paid.2017.06.038

[53] Baron-Cohen S Wheelwright S . The empathy quotient: an investigation of adults with Asperger syndrome or high functioning autism, and normal sex differences. J Autism Dev Disord. (2004) 34:163–75. doi: 10.1023/B:JADD.0000022607.19833.00 15162935

[54] Sparavec A March E Grieve R . The dark triad, empathy, and motives to use social media. Pers Individ Dif. (2022) 194:111647. doi: 10.1016/j.paid.2022.111647

[55] Wakabayashi A Baron-Cohen S Wheelwright S Goldenfeld N Delaney J Fine D . Development of short forms of the Empathy Quotient (EQ-Short) and the Systemizing Quotient (SQ-Short). Pers Individ Dif. (2006) 41:929–40. doi: 10.1016/j.paid.2006.03.017

[56] Muncer SJ Ling J . Psychometric analysis of the empathy quotient (EQ) scale. Pers Individ Dif. (2006) 40:1111–9. doi: 10.1016/j.paid.2005.09.020

[57] Baron-Cohen S . The essential difference: Men women and the extreme male brain. London: Penguin Books (2003).

[58] Jolliffe D Farrington DP . Development and validation of the basic empathy scale. J adolescence. (2006) 29:589–611. doi: 10.1016/j.adolescence.2005.08.010 16198409

[59] Puthillam A Karandikar S Kapoor H . I see how you feel: How the dark triad recognizes emotions. Curr Psychol. (2021) 40:3966–73. doi: 10.1007/s12144-019-00359-x

[60] Cabedo-Peris J Marti-Vilar M Merino-Soto C Ortiz-Moran M . Basic empathy scale: A systematic review and reliability generalization meta-analysis. Healthcare. (2021) 10:29.doi: 10.3390/healthcare10010029 35052193 PMC8775461

[61] Reniers RL Corcoran R Drake R Shryane NM Völlm BA . The QCAE: A questionnaire of cognitive and affective empathy. J Pers Assess. (2011) 93:84–95. doi: 10.1080/00223891.2010.528484 21184334

[62] Mehrabian A Epstein N . A measure of emotional empathy. J Pers. (1972) 40:525–43. doi: 10.1111/j.1467-6494.1972.tb00078.x 4642390

[63] Vachon DD Lynam DR . Fixing the problem with empathy: Development and validation of the affective and cognitive measure of empathy. Assessment. (2016) 23:135–49. doi: 10.1177/1073191114567941 25612628

[64] Lima FFD Osório FDL . Empathy: assessment instruments and psychometric quality–a systematic literature review with a meta-analysis of the past ten years. Front Psychol. (2021) 12:781346. doi: 10.3389/fpsyg.2021.781346 34899531 PMC8653810

[65] Spreng RN McKinnon MC Mar RA Levine B . The Toronto Empathy Questionnaire: Scale development and initial validation of a factor-analytic solution to multiple empathy measures. J Pers Assess. (2009) 91:62–71. doi: 10.1080/00223890802484381 19085285 PMC2775495

[66] Davis MH . Interpersonal Reactivity Index (IRI). Washington, DC: American Psychological Association (1980). doi: 10.1037/t01093-000

[67] Zhang F-f Dong Y Wang K . Reliability and validity of the Chinese version of the Interpersonal Reactivity Index -C. Chin J Clin Psychol. (2010) 18:155–7. doi: 10.16128/j.cnki.1005-3611.2010.02.019

[68] Dragostinov Y Mõttus R . Test-retest reliability and construct validity of the brief Dark Triad measurements. J Pers Assess. (2023) 105:143–8. doi: 10.1080/00223891.2022.2052303 35377780

[69] Jonason PK Webster GD . The dirty dozen: A concise measure of the dark triad. psychol Assess. (2010) 22:420–32. doi: 10.1037/a0019265 20528068

[70] Pechorro P Jonason PK Raposo V Maroco J . Dirty Dozen: A concise measure of Dark Triad traits among at-risk youths. Curr Psychol. (2021) 40:3522–31. doi: 10.1007/s12144-019-00288-9

[71] Raskin R Terry H . A principal-components analysis of the Narcissistic Personality Inventory and further evidence of its construct validity. J Pers Soc Psychol. (1988) 54:890. doi: 10.1037/0022-3514.54.5.890 3379585

[72] del Rosario PM White RM . The Narcissistic Personality Inventory: Test–retest stability and internal consistency. Pers Individ Dif. (2005) 39:1075–81. doi: 10.1016/j.paid.2005.08.001

[73] Emmons RA . Narcissism: theory and measurement. J Pers Soc Psychol. (1987) 52:11–7. doi: 10.1037/0022-3514.52.1.11 3820065

[74] Brown RP Zeigler-Hill V . Narcissism and the non-equivalence of self-esteem measures: a matter of dominance? J Res Pers. (2004) 38:585–92. doi: 10.1016/j.jrp.2003.11.002

[75] Barelds DP Dijkstra P . Narcissistic personality inventory: Structure of the adapted Dutch version. Scandinavian J Psychol. (2010) 51:132–8. doi: 10.1111/j.1467-9450.2009.00737.x 19614906

[76] Jones DN Paulhus DL . Machiavellianism. In: Leary MR Hoyle RH , editors. Handbook of individual differences in social behavior. New York: Guilford Press (2009). p. 102–20.

[77] Gu H Wen Z Fan X . Structural validity of the Machiavellian Personality Scale: A bifactor exploratory structural equation modeling approach. Pers Individ Dif. (2017) 105:116–23. doi: 10.1016/j.paid.2016.09.042

[78] Levenson MR Kiehl KA Fitzpatrick CM . Assessing psychopathic attributes in a noninstitutionalized population. J Pers Soc Psychol. (1995) 68:151. doi: 10.1037/0022-3514.68.1.151 7861311

[79] Barry CT Frick PJ Killian AL . The relation of narcissism and self-esteem to conduct problems in children: A preliminary investigation. J Clin Child Adolesc Psychol. (2003) 32:139–52. doi: 10.1207/S15374424JCCP3201_13 12573939

[80] Paulhus DL Neumann CS Hare RD . The SRP-4: Self-report psychopathy scale. 4th. Toronto, ON: Multi-Health Systems (2016).

[81] Laliberté E Laliberté ME . Package ‘Metacor’, Version 1.0-2.1. Vienna, Austria: The R Foundation (2009).

[82] Biggerstaff BJ Tweedie RL . Incorporating variability in estimates of heterogeneity in the random effects model in meta-analysis. Stat Med. (1997) 16:753–68. doi: 10.1002/(SICI)1097-0258(19970415)16:7<753::AID-SIM494>3.0.CO;2-G 9131763

[83] Higgins JP Thompson SG . Quantifying heterogeneity in a meta-analysis. Stat Med. (2002) 21:1539–58. doi: 10.1002/sim.1186 12111919

[84] Khodabakhsh MR Besharat MA . Mediation effect of narcissism on the relationship between empathy and the quality of interpersonal relationships. Procedia-Social Behav Sci. (2011) 30:902–6. doi: 10.1016/j.sbspro.2011.10.175

[85] Gojković V Dostanić JS Đurić V . Structure of darkness: the dark triad, the ‘dark’empathy and the ‘dark’narcissism. Primenjena psihologija. (2022) 15:237–68. doi: 10.19090/pp.v15i2.2380

[86] Barry C Kauten R Lui JHL . Self-perceptions of social support and empathy as potential moderators in the relation between adolescent narcissism and aggression. Individ Dif Res. (2014) 12:170–9.

[87] Ash S Greenwood D Keenan JP . The neural correlates of narcissism: is there a connection with desire for fame and celebrity worship? Brain Sci. (2023) 13:1499. doi: 10.3390/brainsci13101499 37891865 PMC10605183

[88] di Giacomo E Andreini E Lorusso O Clerici M . The dark side of empathy in narcissistic personality disorder. Front Psychiatry. (2023) 14:1074558. doi: 10.3389/fpsyt.2023.1074558 37065887 PMC10097942

[89] Rogoza R Zemojtel-Piotrowska M Campbel WK . Measurement of narcissism: From classical applications to modern approaches. Studia Psychologica (2018) 18:27–48. doi: 10.21697/sp.2018.18.1.02

[90] Al Aïn S Carré A Fantini-Hauwel C Baudouin JY Besche-Richard C . What is the emotional core of the multidimensional Machiavellian personality trait? Front Psychol. (2013) 4:454. doi: 10.3389/fpsyg.2013.00454 23885245 PMC3717508

